# Proposed Pay-Wards at Charing Cross Hospital

**Published:** 1913-04-12

**Authors:** 


### PROPOSED PAY-WARDS AT CHARING CROSS HOSPITAL.

One of the secrets of successful appeal-writing is to ask for something the need of which the public can understand, or, failing that, to use a popular form of expenditure as a stalking-horse for obtaining subscriptions for purposes which to the uninstructed layman may seem a little dull. An example of the former method has been adopted of late by St. John's Hospital for Diseases of the Skin, which, as we have previously noted, is appealing in the papers for a milligram of radium. As first in the field with this new method we hope that Mr. G. A. Arnaudin is reaping the benefits of an idea which, presumably, was born in the secretary's office. Now the Earl of Lonsdale lends weight to his appeal on behalf of Charing Cross Hospital by stating that the improvements contemplated, if £70,000 be subscribed in response, include the opening of a free children's ward and the re-opening as pay wards of the five wards hitherto closed. It would be invidious and beside the point to speculate in which of the two named improvements Mr. Walter Alvey and his board are most interested. But institutional workers will not fail to note with interest the proposed introduction of five pay wards into a voluntary hospital. "The proposed pay wards," we read, "are for those who are unwilling to accept any form of charity, yet are quite unable to afford surgeons' fees and nursing-home expenses. They are sure to prove a boon to thousands of sufferers possessed of only moderate incomes, as the small fee charged—viz. two guineas per week, including operations—will come more easily within their means." That is the propcsal in a nutshell, and it is to be noted that the fee suggested is that mentioned by ourselves last week in discussing the Horsham Cottage Hospital's plan for admitting paying patients.

The admission of paying patients to voluntary hospitals is one which bristles with debatable points, because while all expert administrators are convinced of the ultimate necessity of eventually adopting in this country a classified system which will find a place in our hospitals and Poor-Law infirmaries for every economic class of patient, such a system at present can hardly be said to exist. The proposal in connection with Charing Cross Hospital comes, however, as a most interesting contribution to the discussion of a reconstruction of our whole system of hospital work on co-operative lines, which we developed in detail last week from the point of view of the Poor-Law infirmary. From the voluntary hospital's standpoint the question at first presents itself in the form of a need to supply a connecting link between the free system of admission and the expensive luxury of the modern nursing home. We have at present the Poor-Law infirmary, theoretically still confined to the destitute, but practically admitting many cases, such as septic cases, from which the voluntary hospital is anxious to be free; the voluntary hospital, which caters for those who can support themselves when in health but have to rely on charitable aid when the bread-winner is overtaken by illness; and the nursing home. Into this not well co-ordinated system has now been thrust by legislation the insured person, that is to say, the patient for whose doctor's bill the State is supposed to be responsible, and who has to turn in cases where any specially skilled treatment is required to the voluntary hospital, since institutional treatment was not provided by the National Insurance Act.

The time, then, is ripe for new developments, and Charing Cross Hospital, if the success of its appeal allows its proposal of pay wards to be adopted, is attempting to undertake the economic classification of patients on its own account. There is little doubt that the fee of two guineas, including operations, approximately meets the case of the person who cannot afford the nursing home, while at the same time protecting the hospital from the charge of placing charitably supported institutions in competition with commercial undertakings. On these grounds the appeal has peculiar claims to the support of the public, which we hope will recognise the attempt here conditionally promised to cater for a deserving class of patient hitherto largely unprovided for.

